# Aero Grade Epoxy Vitrimer towards Commercialization

**DOI:** 10.3390/polym14153180

**Published:** 2022-08-04

**Authors:** Alaitz Ruiz de Luzuriaga, Nerea Markaide, Asier M. Salaberria, Itxaso Azcune, Alaitz Rekondo, Hans Jürgen Grande

**Affiliations:** 1 CIDETEC, Basque Research and Technology Alliance (BRTA), Po. Miramón 196, 20014 Donostia-San Sebastian, Spain; 2 POLYMAT, University of the Basque Country, UPV/EHU, Avda. Tolosa 72, 20018 Donostia-San Sebastian, Spain

**Keywords:** vitrimer, dynamic covalent chemistry, epoxy resin, disulfide, associative exchange

## Abstract

Traditional crosslinked aero grade epoxy resins have excellent thermal-mechanical properties and solvent resistance, but they cannot be remolded, recycled, or repaired. Vitrimers can be topologically rearranged via an associative exchange mechanism, endowing them with thermoplasticity. Introducing dynamic bonds into crosslinked networks to obtain more sustainable thermosets is currently an interesting research topic. While recent research into vitrimers has indicated many advantages over traditional thermosets, an important shortcoming has been identified: susceptibility to creep at service temperature due to the dynamic bonds present in the network. In addition, designing aero grade epoxy vitrimers (similar to RTM6 resin) still remains a challenge. Herein, low creep aero grade epoxy vitrimer with thermal and mechanical properties similar to those of aero grade epoxy resins and with the ability to be recyclable, repairable, and reprocessable, has been prepared. In this manuscript, we demonstrate that aero grade epoxy vitrimer with reduced creep can be easily designed by the introduction of a certain fraction of permanent crosslinks, without having a negative effect on the stress relaxation of the material. Subsequently, the mechanical and relaxation properties were investigated and compared with those of classical aero grade epoxy resin. A high Tg (175 °C) epoxy vitrimer was obtained which fulfilled all mechanical and thermal specifications of the aero sector. This work provides a simple network design to obtain aero grade epoxy resins with excellent creep resistance at elevated temperatures while being sustainable.

## 1. Introduction

Epoxy resins are one of the most widely used thermoset resins for structural applications due to their high mechanical properties, chemical resistance to many solvents, high glass transition temperature (Tg) and resistance to creep at elevated temperatures [1,2]. However, due to their thermoset nature, epoxy resins cannot be recycled, reprocessed, or dissolved after cure, making them less environmentally friendly than thermoplastics and generating a lot of waste when parts reach their end of life. At the same time, thermosets present long-curing times, limiting their production to low-medium volume series, in sectors where there is an increasing demand for such materials. The most common disposal solutions for thermoset resin and composites are pyrolysis and landfilling, carrying serious environmental and economic issues [3,4,5,6,7,8,9].

In this sense, in the last 10 years, new approaches aim to convert thermoset resins into dynamic materials by introducing dynamic covalent bonds [10] in the network The introduction of such dynamic covalent bonds permits the obtention of materials with functional characteristics such as self-healing, recyclability, repairability, reprocessability, shape memory, and adaptability [10,11,12,13,14,15,16,17]. The introduction of dynamic covalent bonds in each crosslinking point of the network leads to the obtention of the covalent adaptable network (CAN), a new concept introduced in polymer chemistry by Bowman and others [18,19,20,21,22,23,24,25,26]. CANs contain an exchangeable covalent bond at each crosslinking point that can be rearranged thermally (or under another stimulus) via a dissociative or associative mechanism. Dissociative bonds rely on a triggered displacement of the association equilibrium towards the endothermic dissociated state, decreasing the crosslinking density and thus, obtaining a thermoplastic-like material. This decrease in crosslink density leads to a sudden viscosity drop that allows rapid reprocessing but also uncontrolled deformation at high temperatures and worse solvent resistance. On the other hand, associative networks maintain a constant crosslink density even at high temperatures because a new covalent bond is formed simultaneously or before breaking an existing covalent bond. The first reworkable epoxy network was developed by Leibler and coworkers [27]. They discovered that crosslinked epoxy networks can undergo transesterification exchange with the addition of a zinc catalyst.

Despite the good properties associated with vitrimers, these materials have an Achilles’ heel: the possible creep at use conditions. Creep is the continuous, time-dependent deformation of a material under a certain load and temperature [28]. While stress relaxation of vitrimers at high temperatures allows them to be malleable and reprocessable, creep at high temperatures is highly undesirable as it prevents the use of these reprocessable networks for many applications where high performance is required. This creep behavior of some vitrimer materials could be one of the main drawbacks to replacing the traditional thermoset for high-performance applications such as in the aerospace industry. In our previous study, we demonstrated that the careful selection of dynamic bonds and dynamic bond exchange kinetics could suppress the creep of aromatic disulfide-containing vitrimers below their glass transition (Tg) or vitrimer temperature (Tv) [29]. However, some groups have demonstrated that substantial creep can occur in some systems below the Tg or Tv [30,31]. Thus, there is still the need to design vitrimer materials with reduced creep at high temperatures. Traditional thermosets exhibit nearly zero creep above their elastic limit at temperatures below the Tg and they show limited creep at temperatures above the Tg in contrary to thermoplastic materials. In this sense, some groups [32,33] have demonstrated that the introduction of some permanent crosslinks in the network is a good approach to reduce the creep in vitrimers while maintaining the capacity to be reprocessed, repaired, and recycled. In order to find a balance between permanent and dynamic crosslinks, the maximum permanent crosslink density to maintain vitrimer properties has been related to the incipient percolation of permanent crosslinks in the network. This incipient percolation of permanent crosslinks is related to the gelation point defined by Flory and Stockmayer [34,35]. Torkelson et al. [32] developed a quantitative theory describing the relationship between the presence of permanent crosslinks in vitrimers and reprocessing behavior. According to Torkelson et al. theory, the critical condition to obtain a reprocessable network, regardless of dynamic chemistry, is to have insufficient permanent crosslinks to create a percolated network. Another approach with the potential to avoid the creep of vitrimers at high temperatures is to design a dynamic network with high activation energy (*Ea*) [17,29,36,37]. The design of a vitrimer with high *Ea* offers high creep resistance a few tens of degrees below the reprocessing temperature, due to the high-temperature dependence of bond exchange. In this sense our group has designed a bunch of disulfide-containing epoxy vitrimers with tailor-made *Ea*, ranging up to 340 kJ/mol [29]. However, this high *Ea* was only achieved in 2-amino phenyldisulfide containing epoxy vitrimers which offer lower Tg and thermal stability than vitrimers containing 4-amino phenyl disulfide.

In the aerospace industry, epoxy resins are difficult to be replaced due to their good thermal and mechanical properties. The qualification process for new aerospace materials can take many years and is extremely expensive. Since Hexcel HexFlow^®^ RTM6 is a widely utilized and aerospace-certified epoxy resin, which has been in service for 20 years, it would be helpful to obtain an epoxy vitrimer with the same mechanical and thermal properties in order to replace it. Everything inside commercial aircraft goes through a process called “certification”. Based on this background the aim of the research described in this paper was to obtain an epoxy resin comparable to HexFlow^®^ RTM6 resin while being reprocessable, reparable, weldable, and recyclable to be commercially available for the aerospace industry. Here, for the first time, a simple and commercially available high Tg epoxy vitrimer ready to be used in the aeronautical sector has been prepared and thoroughly characterized. First, the curing kinetics, as well as thermal and mechanical properties, have been investigated. Secondly, the dynamic properties of the resin have been widely studied by stress relaxation and creep experiments to demonstrate its thermoformability and reprocessability.

## 2. Materials and Methods

### 2.1. Materials

*N*,*N*,*N*′,*N*′-Tetraglycidyl-4,4′-methylenebisbenzenamine (TGMDA) (MY21) and Bisphenol-F diglycidyl ether (DGBF) (PY306) based epoxy resins were purchased from Huntsman Advanced Materials. 4-Aminophenyl disulfide 98% (4-AFD), was purchased from Sigma-Aldrich and was used as received. RTM6 resin was purchased from Hexcel.

### 2.2. Methods

Differential scanning calorimetry (DSC) measurements were performed using a DSC from TA Instruments (Discovery DSC25 Auto controlled by TRIOS software) over a temperature range from 25 °C to 200 °C under nitrogen at a scan rate of of 20 °C min^−1^. The glass transition temperature (Tg) was obtained as the inflection point of the heat flow step. The thermogravimetric measurements (TGA) were performed on a TA Instruments Q500 equipment controlled by TA Universal Analysis software under an air atmosphere at a heating rate of 10 °C min^−1^ from 25 °C to 600 °C. Thermogravimetric isothermal tests were carried out at 200 °C, 210 °C, 220 °C, and 230 °C under an air atmosphere. Thermomechanical experiments were performed using TA Instruments DMA Q800 equipment on rectangular samples (12.5 × 2 × 17.5 mm) on single cantilever beam from 25 to 250 °C. The temperature-dependent behavior was studied by monitoring changes in force and phase angle, keeping the amplitude of oscillation (15 µm) (within a linear viscoelastic region) and frequency (1 Hz) constant at a 3 °C min^−1^ heating rate. Tensile stress-relaxation experiments were also performed on a DMA Q800 instrument controlled by TA Universal Analysis software in tensile mode (6 × 1 × 38 mm). To maintain straightness, samples were initially preloaded at a force of 1 × 10^−3^ N and heated to testing temperature. Before starting with the test, samples were allowed 30 extra minutes to reach the thermal equilibrium. After that, the specimens were stretched by 1% and the deformation was maintained during the test. The decrease in stress over time at each temperature was recorded, and the stress relaxation modulus was calculated.

The relaxation times at each testing temperature were determined from the Maxwell equation, as the time required to relax 63% of the initial stress. With the relaxation times obtained at each temperature, the activation energy *Ea* was calculated, using the Arrhenius equation (Equation (1)):

(1) $$\tau \left(T\right)=\tau_{0\;}\exp\left(\frac{Ea}{RT}\right)$$

From the Arrhenius relation, the topology freezing transition temperature (Tv) was obtained as the temperature at which the material reaches a viscosity of 10^12^ Pa [38].

Creep experiments below the Tg were determined by a DMA Q800 instrument in dual cantilever mode. After soaking for 2 min at the testing temperature, 10 MPa (14% UTS (Ultimate tensile strength)) constant stress was applied for 25 min. Creep experiments above the Tg were determined by a DMA Q800 instrument in tensile mode. After soaking for 2 min at each testing temperature, 1 MPa constant stress was applied. The strain change was monitored over time. For the creep recovery experiment, the constant stress of 1 MPa was applied at 210 °C and 215 °C for 120 min, and a recovery step of 90 min was applied.

Rheological characterization was performed using an AR2000ex rheometer controlled by TA Data Analysis software from TA instruments using a 25 mm plate-plate geometry and a gap of 1 mm. First, strain sweep experiments were performed to determine the linear viscoelastic regimen (LVR) of the materials. Time sweep experiments were carried out from 80 °C to 130 °C,) to determine gel point as the crossover of G′ and G″ (1% of strain and 1 Hz).

Mechanical characterization of both epoxy resins was performed using an INSTRON 3365 Long travel Elastomeric Extensometer controlled by Bluehill Lite software. Tensile strength measurements were carried out according to UNE-EN-ISO 527 standard, using dumbbell-type test specimens at an elongation rate of 1 mm·min^−1^ and flexural tests were performed according to ISO 178 standard.

The reprocessing capacity of cured aero grade vitrimer was studied using 120 × 60 × 2 mm specimens. First, the sample was preheated at 200 °C for 5 min and then the preheated sample was placed between two Teflon-coated metal plates. The reprocessing of the sample was carried out on a VOGT hot press LABO PRESS 200T at 200 °C and 20 bar for 5 min. The demoulding of the sample was performed after cooling down below the Tg of the aero grade epoxy vitrimer.

### 2.3. Synthesis and Characterization

The synthesis of the aero grade epoxy vitrimer used in the present study was performed following the previously reported method by our group [17]. Briefly, TGMDA and DGBF resins were mixed (MY721, 44 g; PY306, 56 g) and degassed at 80 °C. Once the resin mixture was degassed, 4-AFD (50 g) was added and mixed by heating at 80 °C and degassed under vacuum. Then, the resulting viscous liquid was poured between two glass plates separated with a 4 mm, 2 mm or 1 mm silicon joint and cured in an oven at 130 °C for 1 h and post-cured at 180 °C for 0,5 h. The curing reaction was followed by FTIR, where complete curing was confirmed by the disappearance of the epoxide bands at 3056 and 915 cm^−1^ (Figure S1). Complete curing of the aero grade epoxy vitrimer was confirmed by differential scanning calorimetry (DSC) where no residual curing exothermic peak was observed (Figure S2).

## 3. Results and Discussion

**Figure 1:**
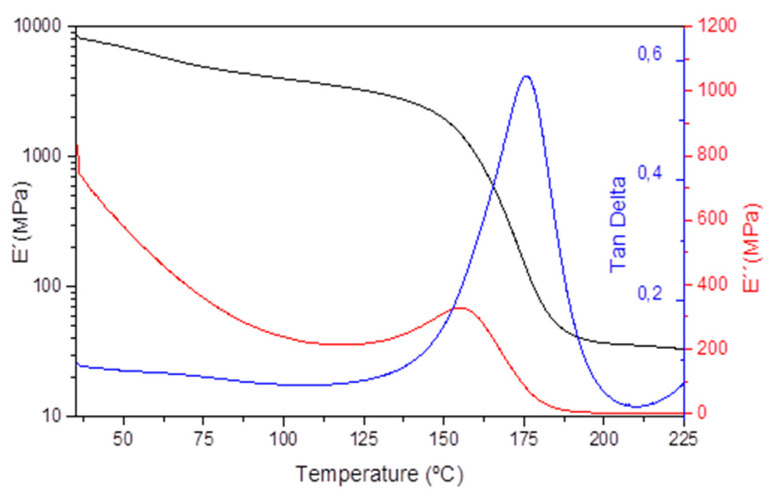
DMA curve obtained for epoxy vitrimer, representing storage modulus (E′), loss modulus (E″), and tan delta versus temperature. Tg = 176 °C was determined from the maximum of tan delta.

Our group previously demonstrated that repair, reprocessing, and recycling could be effectively achieved in medium Tg epoxy thermosets (130 °C) [17]. However, these resins are not useful because of their low Tg for the aeronautics sector due to the high creep at high temperatures (120 °C). In order to obtain a high Tg epoxy resin for the aero sector with reduced creep at high temperatures and to minimize the associated detrimental effects, in this work we have investigated the effect of incorporating irreversible crosslinks. For the synthesis of high Tg (>170 °C) epoxy resin, we used a mixture of tetrafunctional (TGMDA), and bifunctional (DGBF) epoxy resins crosslinked with dynamic disulfide containing amine hardener (4-AFD). The cured epoxy vitrimer showed a Tg of 172 °C measured by DSC. The cross-linking of the high Tg epoxy resin was proven by monitoring the storage modulus (E′) over temperature. A rubbery plateau of 40 MPa at 200 °C, typically attributed to chemically crosslinked polymer systems, confirmed the presence of a 3D network (Figure 1). The obtained aero grade epoxy resin has been compared with reference RTM6 epoxy resin (Figure S3). The crosslink density (ν_C_) provides an idea of the network structure. According to the theory of rubber elasticity, ν_C_ can be experimentally obtained using Equation (2).

ν_C_ = E′_r_/3 A R T (2)

where E’_r_ is the rubbery plateau at T g +  30 °C, A is the front factor often assumed to be unity, R is the gas constant (8.314 J·mol^−1^·K^−1^) and T is the absolute temperature in K. The average molecular weight between crosslinks (M_C_) can be also calculated from Equation (3).

M_C_ = d/ν_C_ (3)

where d is the cured resin density.

Table 1 shows the crosslinking density and Mc of both aero grade epoxy resin and references RTM6 resin.

As can be seen in Table 1, reference epoxy resin presents higher Tg than developed aero grade epoxy resin. However, the obtained Tg value for the epoxy vitrimer is still valid for the aeronautical sector. The difference in the Mc of both resins could come from the different molecular weights of both epoxy monomers and hardeners.

### 3.1. Thermal Stability

In order to study the thermal stability of prepared aero grade epoxy vitrimer, thermogravimetric measurements were carried out.

**Figure 2:**
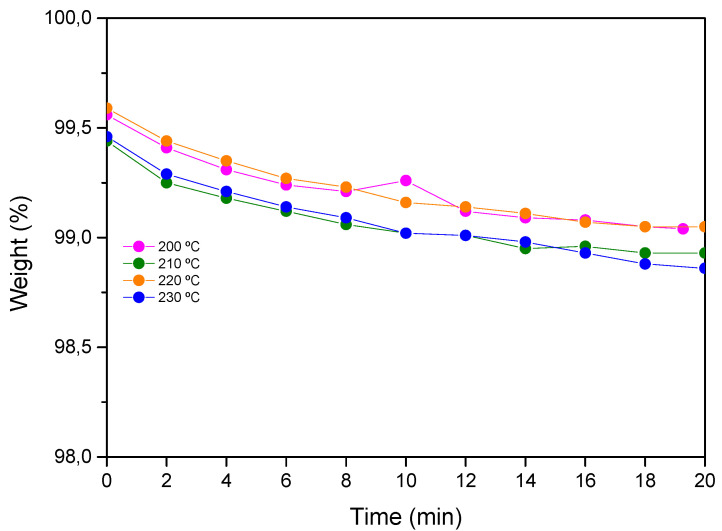
Thermal stability of epoxy vitrimer in air.

The thermogravimetric analysis (TGA) of the cured samples was performed on a TA Instruments Q500 equipment under an air atmosphere at a heating rate of 30 °C·min^−1^ from 25 °C to isothermal conditions. Isotherms were measured under an air atmosphere for 20 min (Figure 2). Additionally, the Tg of the aged samples was measured by DSC in order to study the possible degradation of the resin (Figure S4).

Table 2 shows the results obtained for different temperatures. As can be seen, aero grade epoxy vitrimer shows a weight loss lower than 1.2% after 20 min at temperatures up to 230 °C.

Thermogravimetric analysis showed that developed epoxy vitrimer possesses thermal stability up to 210 °C (weight loss lower than 1% without Tg decrease) when exposed to 20 min under air atmosphere. From these results, it can be concluded that 210–220 °C is the maximum temperature at which the resin can be exposed under air atmosphere to assure no degradation of the epoxy vitrimer matrix. Thermal stability together with dynamic stress relaxation results will permit to the establishment of the time and temperature conditions for future thermoforming processes.

### 3.2. Fracture Toughness

One of the major drawbacks of the RTM6 resin is its inherent brittleness, ascribed to the highly crosslinked network and to the high internal stresses produced by volume shrinkage during curing. With the aim of reducing this brittleness, different approaches have been studied to increase toughness. In this sense, the introduction of a dynamic hardener could be a new approach to increasing the resin toughness. It has been demonstrated that polymer networks formed by transient crosslinks constantly reconfigure and dissipates energy, increasing the toughness of the resin. In developed aero grade epoxy vitrimer, the easier cleavage and rearrangement of aromatic disulfide bonds present in the 3D network, could be beneficial to stimulate the release of internal stresses that occurred in the curing of the network [39].

Fracture toughness tests were performed by using SENB (Single-Edge Notched Bending) specimens in a 3-point bending configuration, according to the ISO 13586 test standard. Pre-notches were machined to a depth of 0.45 times the specimen width using a CEAST notching machine (Notchvis 6951.000, Turin, Italy). Subsequently, the pre-notches were sharpened using a femtosecond laser (femtolaser) to a depth of approximately 0.5 times the width of the specimen. This process allowed the production of SENB specimens for 3-point bending. The SENB specimens were tested on a universal testing machine (SUN2500, Galdabini, Italy) with a support spacing of 48 mm and a crosshead displacement speed of 1 mm/min.

As can be seen from the force-displacement graphs in Figure S5, the mechanical response of all specimens was linear and elastic up to fracture. The non-linearity and the observed variation in the displacement is due to pin penetration and compression effects. Therefore, following the guidelines set out in ISO 13586, the stress intensity factor *K_Q_* can be calculated using Equation (4),

(4) $$K_{Q}=f\frac{F_{Q}}{BW^{1/2}}$$

where F*_Q_* is the maximum force, *B* is the thickness of the specimen, *W* is the width of the specimen and *f* is a geometrical calibration factor. The results of the *K_Q_* calculation are shown in Table S1.

There are two main parameters that describe the fracture toughness of a material: K_1C_, the critical stress intensity factor near the edge of a crack, and G_1C_, which is the energy needed to create two surfaces during the fracture propagation. Table 3 reports the fracture toughness test results for epoxy vitrimer resin and RTM6 neat resin. It is observed that, for the epoxy vitrimer, the value of K_1C_ is improved from 0.62 Mpa·m^1/2^ to 1.02 MPa·m^1/2^, which corresponds to an increase of 64%. The increase in the fracture toughness of developed aero grade epoxy vitrimer can be attributable to the presence of dynamic disulfide bonds in the matrix together with lower Tg of the resin compared to RTM6 resin. Figures S6 and S7 show the fracture surfaces of tested specimens where smooth fracture surfaces can be observed. No difference was seen in the fracture surfaces of the tested samples.

### 3.3. Rheology and Curing Kinetics

In order to study the processability of the 3R epoxy resin for the RTM process, the rheology of uncured resin was characterized. Taking into account the specifications for the RTM process for uncured resin, three different parameters were measured (initial viscosity at different temperatures, the time needed to reach 200 mPa·s at different temperatures, and time needed to achieve the gel point. Gel point was defined as the crossover point of the elastic modulus (G′) and loss modulus (G″)) (Figures S8 and S9).

In Table 4, a summary of the obtained results is shown.

To study the effect of curing time at different temperatures a DSC analysis was carried out. The degree of cure at each temperature after a temperature ramp of 30 °C/min was calculated. Dynamic curing of both aero grade epoxy vitrimer and reference RTM6 was also performed in order to see the differences both in the curing exotherm and curing temperature (Figure S10). Table S2 shows the curing enthalpy of both systems as well as exothermic peak temperature. According to the obtained data, both systems present similar total heat of reaction. However, the exothermic peak temperature is around 40 °C lower for aero grade epoxy vitrimer which suggested that the chemical reactivity of 4-AFD (dynamic hardener) is higher than the reference.

**Figure 3:**
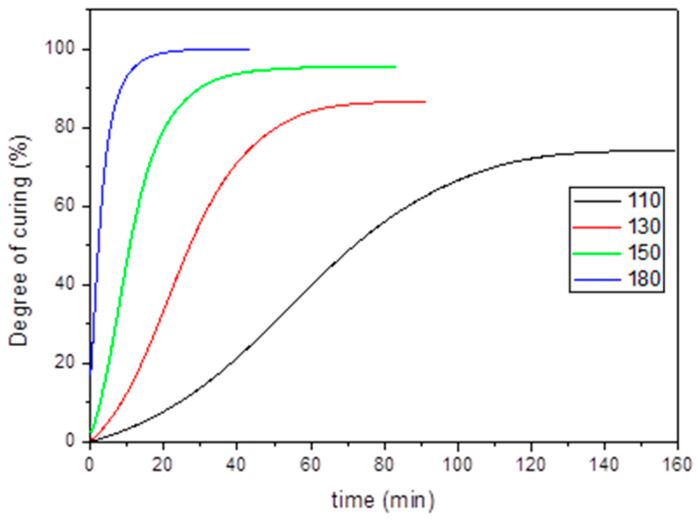
Curing degree of aero grade epoxy vitrimer at different temperatures.

The heat of cure can be used to quantitatively determine the degree of curing of the resins. In fact, the degree of cure is defined as the ratio of the released heat up to a certain time by the total heat of the reaction. It ranges from 0, for uncured resin, to 100%, for completely cured resin. In Figure 3 the extent of curing at each temperature vs. time can be observed.

### 3.4. Mechanical Properties

**Figure 4:**
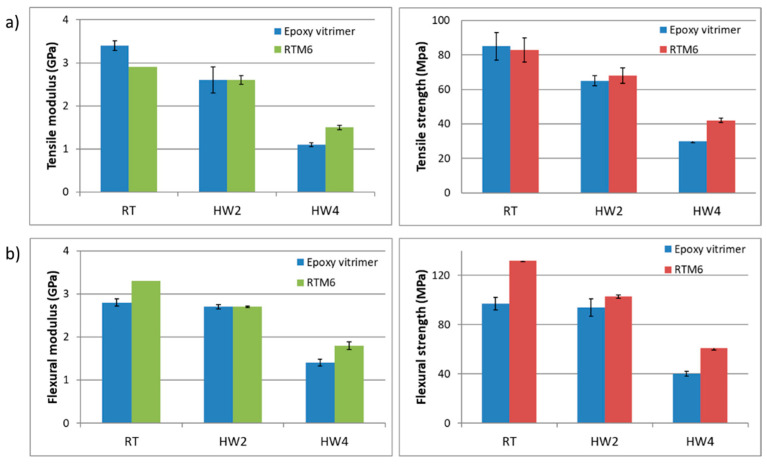
(**a**) Tensile modulus and tensile strength of epoxy vitrimer and RTM6 formulations at RT, HW2, and HW4. (**b**) Flexural modulus and flexural strength of epoxy vitrimer and RTM6 formulations at RT, HW2, and HW4.

Tensile and flexural tests were performed to characterize the mechanical properties of aero grade epoxy vitrimer and to compare it with reference RTM6 epoxy resin (Figure 4).

Considering that developed epoxy vitrimer is focused on the aeronautical sector it is crucial to validate the mechanical properties in hot/wet conditions. Aircraft components manufactured from composite materials are typically exposed to hygrothermal environments which are well known to alter the physical and mechanical properties of fiber-reinforced composites. Epoxy resins are hydrophilic, which means that they absorb moisture from the environment. This makes epoxy resins susceptible to high water absorption. It is well known and studied that absorbed moisture lowers the mechanical properties of the materials. Water absorption into epoxy resins changes both chemical and physical properties through different mechanisms such as plasticization, crazing, hydrolysis, and swelling. Plasticization is the most important physical change that occurs due to the interaction of the water molecules with polar groups present in the epoxy resins and thus, decreasing the Tg. In order to take all these into account, the mechanical and thermal analysis of water absorbed samples has been also carried out. Both developed epoxy vitrimer and reference RTM6 were characterized at three different conditions:

1. Dry/room temperature.
2. HW2: Aged 14 days at 70 °C and 85% RH (relative humidity) and tested at 70 °C.
3. HW4: Aged 14 days at 70 °C and 85% RH and tested at 120 °C.

Water uptake measurements were carried out according to UNE-EN-ISO 4611. During the period of the aging study, gravimetric measurements were performed on balance. At selected times, different samples were removed from the chamber, dried superficially, and cooled to ambient temperature prior to their characterization. The samples were aged until a constant weight was obtained (Figure S11). It was observed that the water uptake of epoxy vitrimer is similar (2,4%) to the commercial RTM6 resin (2%).

As can be seen in Figure 4, both materials showed similar tensile and flexural properties at *RT* and HW2 conditions, which means that the mechanical properties of both systems are similar at temperatures below Tg. However, if we compare both materials at HW4 conditions, epoxy vitrimer performed lower: −30% in tensile strength and −35% in flexural strength. The observed behavior at 120 °C is related to the Tg decrease of the resins due to water absorption. The water absorbed due to the aging of the samples until saturation decreases the Tg [41], reducing the temperature difference between the Tg and the test temperature and thus, reducing its tensile and flexural strength.

### 3.5. Stress Relaxation

Due to the presence of dynamic hardener, developed aero grade epoxy resin was expected to show vitrimer behavior. The time and temperature-dependent relaxation modulus was tested by DMA to characterize its heat-induced malleability. In the following subsections, stress relaxation was studied first, followed by creep tests to end with thermoforming and reprocessing tests.

**Figure 5:**
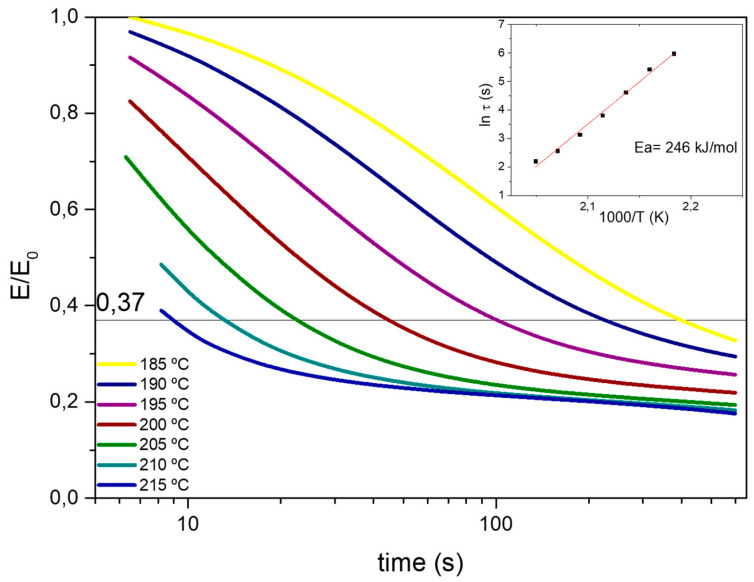
Normalized stress relaxation curves at different temperatures for aero grade epoxy vitrimer (Inset). Calculated activation energy (*Ea*) from stress relaxation test.

During the stress relaxation test, in order to assure the straightness of the specimens, the samples were preloaded by applying 10^−3^ N.. After reaching the required temperature, 1% strain was applied and the relaxation modulus was monitored over time. In Figure 5 the normalized stress relaxation curves at different temperatures are shown. As can be seen in Figure 5, at temperatures above Tg, the epoxy vitrimer was able to relax stress and flow. The relaxation time (τ*) at each temperature was determined from Maxwell model for viscoelastic fluids as the time required to relax 63% of the initial stress [28]. The relaxation times obtained for the aero grade epoxy vitrimer are similar to previously reported 100% epoxy vitrimer [17] despite having 18.5% of permanent crosslinks (37% of tetrafunctional epoxy monomer, where 50% of the total crosslinks are permanent). It is hypothesized that due to the addition of tetrafunctional epoxy resin, the disulfide concentration increased, leading to faster exchange kinetics. The activation energy of the bond exchange mechanisms of the aero grade epoxy vitrimer was also measured. The temperature dependence of τ* was fitted to an Arrhenius relation and the activation energy (*Ea*) was calculated. The Arrhenius plot presents a linear correlation of ln (τ) with 1000/T in the measured temperature range (Figure 5 inset). From Equation (1), activation energy (*Ea*) of 246 kJ·mol^−1^ was calculated. Activation energy (*Ea*) values are characteristic of each system and represent the sensitivity of the process to the temperature. For structural materials, high *Ea* is required in order to have high mechanical properties at working temperature while being reprocessable, reparable, and recyclable at temperatures above the Tg. In this sense, to reduce the creep at low temperatures and achieve low reprocessing time at high temperatures, high *Ea* is needed.

From the Arrhenius relation, the topology freezing temperature (Tv) was also obtained. Tv is defined as the temperature at which the material reaches a viscosity of 10^12^ Pa·s [38,42,43,44]. Topology freezing temperature (*T*_v_) was calculated from the Maxwell equation: *η* = *Gp*τ* where *η* is the viscosity, *Gp* is the plateau modulus and τ* is the characteristic relaxation time. Using the equation above, τ* was calculated to be around 10^5^ s. *T*_v_ could be calculated by the extrapolation of the Arrhenius fitted line (Figure 5 inset) when τ* = 10^5^ s. For the aero grade epoxy vitrimer a Tv of 152 °C was calculated, which is below the Tg of the resin. For the present system, once the Tg is overpassed, an exchangeable reaction happens fast and vitrimer can be reprocessed.

### 3.6. Creep

**Figure 6:**
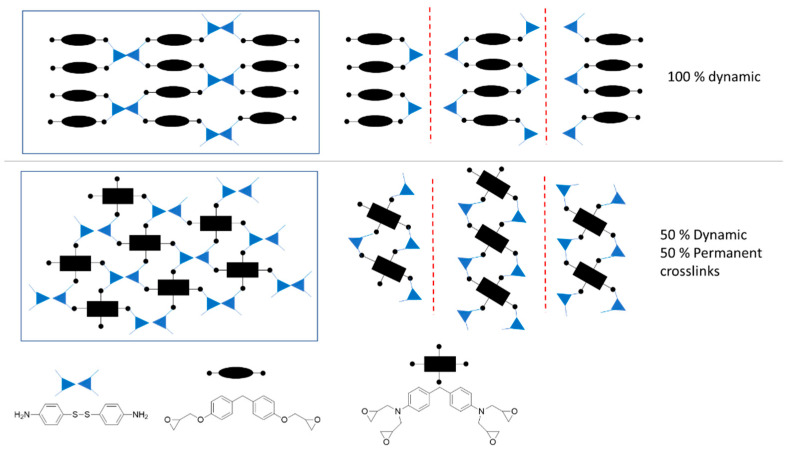
These conceptual schemes represent the dynamic and permanent crosslinks present in aero grade epoxy vitrimer. To simplify, networks with 100% difunctional epoxy monomer (100% dynamic crosslinks) and with 100% tetrafunctional epoxy monomer (50% dynamic crosslinks) are shown separately.

Another important parameter of vitrimers, besides stress-relaxation, is their resistance to creep. Compared to classical thermoset polymers, vitrimers are prone to creep at high temperatures above their Tg or Tv due to the exchange of dynamic bonds, especially if these exchange reactions are fast [37]. However, it is also known that the introduction of some irreversible crosslinks could reduce the creep at high temperatures. This vitrimer contains 37/63% mol ratio of tetrafunctional/bifunctional epoxy monomers which leads to a network with 18.5% of total crosslinks being permanent. In this section, the coexistence of dynamic bonds together with irreversible crosslinks was analyzed by creep tests at different temperatures. In recent studies reported by Torkelson et al. [32] and Du Prez et al. [45] theoretical formulas were reported to calculate the maximum permanent crosslinks allowed in a vitrimer before losing the reprocessing capacity. The introduced theory is based on the reverse Flory- Stockmayer (F-S) gelation theory [35]. This theory was experimentally validated for crosslinked materials. Torkelson et al. validated the theory for systems maintaining reprocessability having up to 40% of permanent crosslinks. For the system validated by Du Prez et al., this value increased up to 70% of permanent crosslinks. The same approximation was performed in the present paper assuming that both primary and secondary amines will react with the epoxy groups and having a 1.15 excess of amine groups. For the assumption, only tetrafunctional epoxy monomer was taken into account because the network formed with difunctional epoxy will be fully dynamic and reprocessable (Figure 6). For the calculation of maximum tetrafunctional epoxy monomer permitted to obtain a recyclable network, the functionality of the amine hardener will be considered as two because half of the crosslinks will be dynamic. For the present system, it was theoretically found that a maximum of 63% of tetrafunctional epoxy resin should be present to maintain reprocessability.

To assess good creep resistance of developed aero grade epoxy resin, creep recovery experiments were carried out at high temperatures and compared with reference RTM6 epoxy resin. The long-term performance of the material was predicted using the time-temperature superposition (TTS) principle (Figure S12).

**Figure 7:**
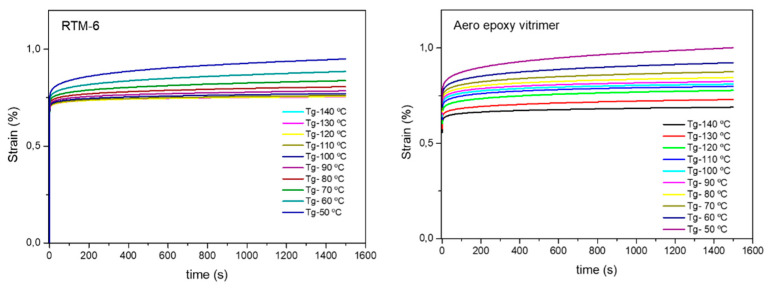
Creep experiments of reference RTM6 and aero grade epoxy vitrimer at temperatures below Tg (from Tg −140 °C to Tg −50 °C) with applied stress of 10 MPa for 25 min.

In order to compare the obtained results, due to the difference in the Tg of the material (175 °C for aero grade epoxy vitrimer and 210 °C for RTM 6 resin), the temperature behavior was normalized to the Tg of each of the materials. As can be seen in Figure 7, it is clearly noticeable that below the Tg (Tg − 50 °C) no creep was observed in both materials. We demonstrate that the designed aero grade epoxy vitrimer is capable of arresting creep at elevated temperatures up to 120 °C. Aero grade epoxy vitrimer exhibited almost no creep with strain values <1% at 120 °C, after 25 min at 10 MPa stress. This excellent creep resistance is comparable to the creep response of conventional crosslinked networks such as RTM6 aero grade epoxy resin.

**Figure 8:**
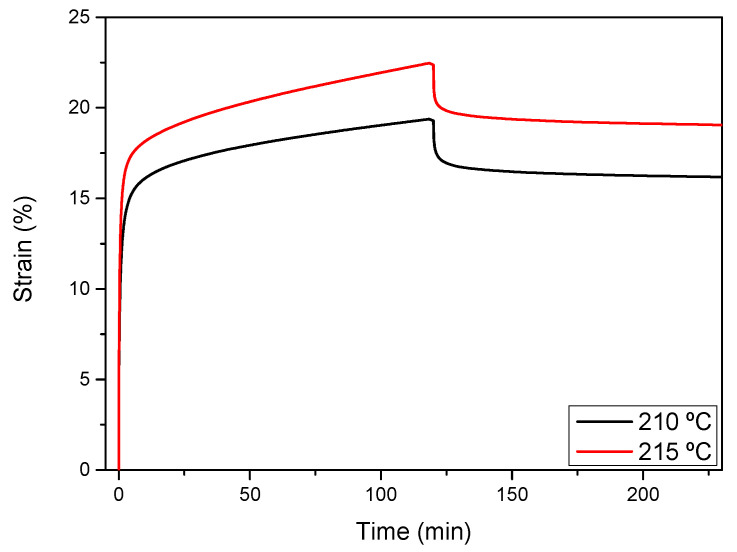
Creep recovery experiments of aero grade vitrimer at different temperatures with an applied stress of 1 MPa.

It is well known that the presence of permanent crosslinks could reduce the creep behavior of the developed aero grade epoxy vitrimer at T > Tg. With the aim to corroborate the heat-induced malleability at temperatures above the Tg, creep experiments were also carried out at 210 °C and 215 °C applying 1 MPa of constant stress. As can be seen in Figure 8, above the Tg of the material, it starts deforming and this deformation increases with the temperature. However, due to the presence of permanent crosslinks introduced into the matrix no complete deformation was achieved. An irreversible deformation of around 85% was achieved, which is in accordance with the relaxation obtained and the 19% of permanent crosslinks present in the network. The same experiment was repeated for RTM6 resin at Tg +50 °C, but even with low applied stress (0.5 MPa), the resin broke due to the absence of dynamic bonds.

### 3.7. Thermoforming and Reprocessing

The dynamic behavior of aero grade epoxy network was exploited to demonstrate the capability to thermoform high Tg epoxy vitrimers.

**Figure 9:**
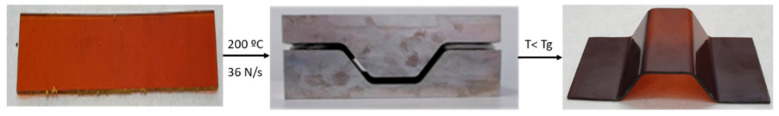
Validation of thermoforming capacity of aero grade epoxy vitrimer.

To validate the thermoforming capacity of developed aero grade epoxy resin, a fully cured 2 mm thick resin plate was hot-pressed in a mold with omega shape. First, the epoxy vitrimer was placed in a pre-heated oven at 200 °C for 5 min together with the mold used for thermoforming. Once the vitrimer and the mold were at the right temperature, the sample was gradually deformed at 36 N/s speed at 200 °C. After the desired deformation was reached, the vitrimer was kept in its new shape to cool down below the Tg to ensure full stress release due to the reorganization of dynamic disulfide bonds. Figure 9 shows the result, confirming the reprocessability of the resin. In order to assure no degradation of the epoxy vitrimer due to the thermoforming process, the Tg of the reprocessed material was measured by DMA (Figure S13). After the thermoforming process, the reshaped material showed a Tg (Tan delta Peak) of 175 °C. No decrease in the Tg of the material was observed after reprocessing confirming no degradation of the vitrimer during the thermoforming process.

This hot-press forming process is very fast compared to actual manufacturing processes for aero parts (autoclave, etc.), thus having a tremendous impact on the aeronautics sector reducing the production time by at least 10 times.

Finally, in order to confirm the reprocessing capabilities, the epoxy vitrimer was grinded to fine powder, which was then hot pressed into a mold by applying heat and pressure (20 bar, 210 °C, 20 min). Figure S14 shows the reprocessed sample and its thermal characterization. The DMA plot of the reprocessed sample was comparable to the initial epoxy vitrimer as the storage modulus showed no changes after reprocessing. Only a small decrease in the Tg was observed due to the lack of compaction in some areas and the possible breakage of non-reversible bonds due to grinding process and high temperature reprocessing.

## 4. Conclusions

Here we showed for the first time that an aero grade aromatic disulfide epoxy vitrimer with reduced creep can be obtained by incorporation of a certain fraction of permanent crosslinks. The epoxy vitrimer exhibited similar properties to widely used aero grade RTM6 aero resin while being reprocessable. The developed aero grade epoxy resin presents an acute temperature response due to the high activation energy (*Ea* = 246 kJ/mol) of the network. This high *Ea* permits the design of resins and composites with high-temperature sensitivity, permitting to arrest of the dynamic behavior up to high temperatures and thus reducing the creep up to the Tg of the material. For the present aromatic disulfide-containing vitrimers, we have determined that up to 30% of permanent crosslinks can be introduced in the network while maintaining reprocessability. Our study provides a promising approach for limiting the non-desirable effects associated with creep in vitrimer applications, particularly for aerospace industry. In addition, the introduction of permanent crosslinks also enhanced the thermal and mechanical properties making the developed aero grade epoxy vitrimer the right candidate for introduction in the aero sector.

## Figures and Tables

**Table 1:** Tan δ, E′, E″, Density, ν_C,_ and M_C_ of aero grade epoxy vitrimer and reference RTM 6 applying rubber elasticity theory.

Ref.	Tan δ Max.	E′ (M Pa) at 25 °C	E′ (M Pa) at 200 °C	Tan δ Peak (°C)	d (g·cm^−3^)	ν_C_ (mol·m^−3^)	M_C_ (g·mol^−1^)
Vitrimer	0.6	9000	40	176	1.27	3390	374
RTM6	1.09	7000	30	218	1.14	2310	494

**Table 2:** Thermal stability of epoxy vitrimer. The initial Tg of the sample and the Tg after isothermal conditions are also shown.

Isotherm Test, Air Atmosphere					
Sample		200 °C	210 °C	220 °C	230 °C
Epoxy vitrimer	W (%) 20’	99.04	98.93	99.05	98.86
	Tg initial	172			
	Tg final	172	173	165	160

**Table 3:** Fracture toughness test results.

Sample	K1c (MPa ·m^1/2^)	G1c (J/m^2^)
Epoxy vitrimer	1.02 ± 0.07	330 ± 40
RTM6 [40]	0.62	114

**Table 4:** Initial viscosity (ƞ0), time to reach 200·mPa·s, and gel time (defined as G′ = G″) at different temperatures.

T (°C)	ƞ_0_ (mPa·s)	t_200mPa·s_ (Min)	t_G__′__=G__″_ (Min)
80	80	152	490
110	21	62	122
130	17	29	47

## Data Availability

Not applicable.

## Supplementary Materials

The following supporting information can be downloaded at: https://www.mdpi.com/article/10.3390/polym14153180/s1, Figure S1: FTIR of uncured (black) and cured aero grade epoxy resin (red); Figure S2: DSC thermogram for aero grade epoxy vitrimer, from where a Tg value of 172 °C was determined; Figure S3: DMA curve obtained for RTM6, representing storage modulus, loss modulus and tan delta versus temperature. Tg = 218 °C was determined from the maximum of tan delta; Figure S4: DSC thermograms of aero grade epoxy vitrimer after aging 20 min in ari atmosphere at 200 °C, 210 °C, 220 °C and 230 °C; Figure S5: Force-displacement graphs of epoxy vitrimer; Figure S6: Fractured surfaces of tested aero grade epoxy vitrimer; Figure S7: Detailed fracture surface analysis after failure. Smooth fracture surface; Figure S8: Viscosity evolution of aero grade epoxy resin at 80 °C, 110 °C and 130 °C; Figure S9: Gel point at 80 °C (a), 110 °C and 130 °C determined as the crossover point G′ = G″; Figure S10: Dynamic DSC curves obtained at 2.5 °C/min for aero grade epoxy vitrimer (blue) and reference RTM6 resin (green); Figure S11: Water uptake of aero grade epoxy vitrimer; Figure S12: Creep strain master curve at 120 °C obtained by horizontal shifting of creep data at different temperatures; Figure S13: DMA curve obtained for thermoformed epoxy vitrimer, representing storage modulus, loss modulus and tan delta versus temperature. Tg = 175 °C was determined from the maximum of tan delta; Fgure S14: (Left) reprocessed aero grade epoxy vitrimer. (Right) DMA plot of reprocessed aero grade epoxy vitrimer; Table S1: Values of the stress intensity factor K_Q_ for all tested materials; Table S2: Enthalpy of cure, Tg of un-cured resin, onset curing temperature and exothermic temperature of aero grade epoxy vitrimer and reference RTM6 resin.

## References

[1] Hu Q., Memon H., Qiu Y., Liu W., Wei Y. (2020). A Comprehensive Study on the Mechanical Properties of Different 3D Woven Carbon Fiber-Epoxy Composites. Materials.

[2] Auvergne R., Caillol S., David G., Boutevin B., Pascault J.-P. (2014). Biobased Thermosetting Epoxy: Present and Future. Chem. Rev..

[3] Pickering S.J. (2006). Recycling technologies for thermoset composite materials—Current status. Compos. Part A Appl. Sci. Manuf..

[4] Pimenta S., Pinho S.T. (2011). Recycling carbon fibre reinforced polymers for structural applications: Technology review and market outlook. Waste Manag..

[5] Yang Y., Boom R., Irion B., van Heerden D.-J., Kuiper P., de Wit H. (2012). Recycling of composite materials. Chem. Eng. Process..

[6] Oliveux G., Dandy L.O., Leeke G.A. (2015). Current status of recycling of fibre reinforced polymers: Review of technologies, reuse and resulting properties. Prog. Mater. Sci..

[7] Li J., Xu P.-L., Zhu Y.-K., Ding J.-P., Xue L.-X., Wang Y.-Z. (2012). A promising strategy for chemical recycling of carbon fiber/thermoset composites: Self-accelerating decomposition in a mild oxidative system. Green Chem..

[8] Wang Y., Cui X., Ge H., Yang Y., Wang Y., Zhang C., Li J., Deng T., Qin Z., Hou X. (2015). Chemical Recycling of Carbon Fiber Reinforced Epoxy Resin Composites via Selective Cleavage of the Carbon–Nitrogen Bond. ACS Sustain. Chem. Eng..

[9] Deng T., Liu Y., Cui X., Yang Y., Jia S., Wang Y., Lu C., Li D., Cai R., Hou X. (2015). Cleavage of C–N bonds in carbon fiber/epoxy resin composites. Green Chem..

[10] Jin Y., Yu C., Denman R.J., Zhang W. (2013). Recent advances in dynamic covalent chemistry. Chem. Soc. Rev..

[11] Jin Y., Lei Z., Taynton P., Huang S., Zhang W. (2019). Malleable and Recyclable Thermosets: The Next Generation of Plastics. Matter.

[12] Roy N., Bruchmann B., Lehn J.-M. (2015). DYNAMERS: Dynamic polymers as self-healing materials. Chem. Soc. Rev..

[13] Kloxin C.J., Scott T.F., Adzima B.J., Bowman C.N. (2010). Covalent Adaptable Networks (CANs): A Unique Paradigm in Cross-Linked Polymers. Macromolecules.

[14] Maeda T., Otsuka H., Takahara A. (2009). Dynamic covalent polymers: Reorganizable polymers with dynamic covalent bonds. Prog. Polym. Sci..

[15] Wojtecki R.J., Meador M.A., Rowan S.J. (2011). Using the dynamic bond to access macroscopically responsive structurally dynamic polymers. Nat. Mater..

[16] Rekondo A., Martin R., Ruiz de Luzuriaga A., Cabanero G., Grande H.J., Odriozola I. (2014). Catalyst-free room-temperature self-healing elastomers based on aromatic disulfide metathesis. Mater. Horiz..

[17] Ruiz de Luzuriaga A., Martin R., Markaide N., Rekondo A., Cabañero G., Rodríguez J., Odriozola I. (2016). Epoxy resin with exchangeable disulfide crosslinks to obtain reprocessable, repairable and recyclable fiber-reinforced thermoset composites. Mater. Horiz..

[18] Chen X., Dam M.A., Ono K., Mal A., Shen H., Nutt S.R., Sheran K., Wudl F. (2002). A Thermally Re-mendable Cross-Linked Polymeric Material. Science.

[19] Denissen W., Rivero G., Nicolay R., Leibler L., Winne J.M., Du Prez F.E. (2015). Vinylogous Urethane Vitrimers. Adv. Funct. Mater..

[20] Bowman C.N., Kloxin C.J. (2012). Covalent Adaptable Networks: Reversible Bond Structures Incorporated in Polymer Networks. Angew. Chem. Int. Ed..

[21] Guerre M., Taplan C., Winne J.M., Du Prez F.E. (2020). Vitrimers: Directing chemical reactivity to control material properties. Chem. Sci..

[22] Azcune I., Odriozola I. (2016). Aromatic disulfide crosslinks in polymer systems: Self-healing, reprocessability, recyclability and more. Eur. Polym. J..

[23] Erice A., Ruiz de Luzuriaga A., Matxain J.M., Ruiperez F., Asua J.M., Grande H.-J., Rekondo A. (2018). Reprocessable and recyclable crosslinked poly(urea-urethane)s based on dynamic amine/urea exchange. Polymer.

[24] Lu Y.-X., Tournilhac F., Leibler L., Guan Z. (2012). Making Insoluble Polymer Networks Malleable via Olefin Metathesis. J. Am. Chem. Soc..

[25] Taynton P., Yu K., Shoemaker R.K., Jin Y., Qi H.J., Zhang W. (2014). Heat- or Water-Driven Malleability in a Highly Recyclable Covalent Network Polymer. Adv. Mater..

[26] Yang Y., Xu Y., Ji Y., Wei Y. (2021). Functional epoxy vitrimers and composites. Prog. Mater. Sci..

[27] Montarnal D., Capelot M., Tournilhac F., Leibler L. (2011). Silica-Like Malleable Materials from Permanent Organic Networks. Science.

[28] Sperling L.H. (2006). Introduction to Physical Polymer Science.

[29] Ruiz de Luzuriaga A., Solera G., Azcarate-Ascasua I., Boucher V., Grande H.-J., Rekondo A. (2022). Chemical control of the aromatic disulfide exchange kinetics for tailor-made epoxy vitrimers. Polymer.

[30] Yang Z., Wang Q., Wang T. (2016). Dual-Triggered and Thermally Reconfigurable Shape Memory Graphene-Vitrimer Composites. ACS Appl. Mater. Interfaces.

[31] Capelot M., Unterlass M.M., Tournilhac F., Leibler L. (2012). Catalytic Control of the Vitrimer Glass Transition. ACS Macro Lett..

[32] Li L., Chen X., Jin K., Torkelson J.M. (2018). Vitrimers Designed Both to Strongly Suppress Creep and To Recover Original Cross-Link Density after Reprocessing: Quantitative Theory and Experiments. Macromolecules.

[33] Cash J.J., Kubo T., Dobbins D.J., Sumerlin B.S. (2018). Maximizing the symbiosis of static and dynamic bonds in self-healing boronic ester networks. Polym. Chem..

[34] Flory P.J. (1941). Molecular Size Distribution in Three Dimensional Polymers. I. Gelation1. J. Am. Chem. Soc..

[35] Stockmayer W.H. (1943). Theory of Molecular Size Distribution and Gel Formation in Branched-Chain Polymers. J. Chem. Phys..

[36] Liu Y., Tang Z., Wang D., Wu S., Guo B. (2019). Biomimetic design of elastomeric vitrimers with unparalleled mechanical properties, improved creep resistance and retained malleability by metal–ligand coordination. J. Mater. Chem. A.

[37] Li L., Chen X., Jin K., Rusayyis M.B., Torkelson J.M. (2021). Arresting Elevated-Temperature Creep and Achieving Full Cross-Link Density Recovery in Reprocessable Polymer Networks and Network Composites via Nitroxide-Mediated Dynamic Chemistry. Macromolecules.

[38] Denissen W., Winne J.M., Du Prez F.E. (2016). Vitrimers: Permanent organic networks with glass-like fluidity. Chem. Sci..

[39] Tsai H.-Y., Nakamura Y., Hu W.-H., Fujita T., Naito M. (2021). Mechanochromism of dynamic disulfide bonds as a chromophoric indicator of adhesion strength for epoxy adhesive. Mater. Adv..

[40] Zotti A., Zuppolini S., Borriello A., Zarrelli M. (2019). Thermal Properties and Fracture Toughness of Epoxy Nanocomposites Loaded with Hyperbranched-Polymers-Based Core/Shell Nanoparticles. Nanomaterials.

[41] Delasi R., Whiteside J. (1978). Effect of Moisture on Epoxy Resins and Composites.

[42] Dyre J.C. (2006). Colloquium: The glass transition and elastic models of glass-forming liquids. Rev. Mod. Phys..

[43] Angell C.A. (1995). Formation of Glasses from Liquids and Biopolymers. Science.

[44] Ediger M.D., Angell C.A., Nagel S.R. (1996). Supercooled Liquids and Glasses. J. Phys. Chem..

[45] Spiesschaert Y., Guerre M., De Baere I., Van Paepegem W., Winne J.M., Du Prez F.E. (2020). Dynamic Curing Agents for Amine-Hardened Epoxy Vitrimers with Short (Re)processing Times. Macromolecules.

