# Supplementary material for: Aero Grade Epoxy Vitrimer towards Commercialization
Source: Polymers (Basel). 2022 Aug 4;14(15):3180. doi: 10.3390/polym14153180 (PMC9371194; doi:10.3390/polym14153180)
Supplement: Supplementary file 1 [file polymers-14-03180-s001.zip › polymers-1826913-supplementary.pdf]

Supplementary Materials

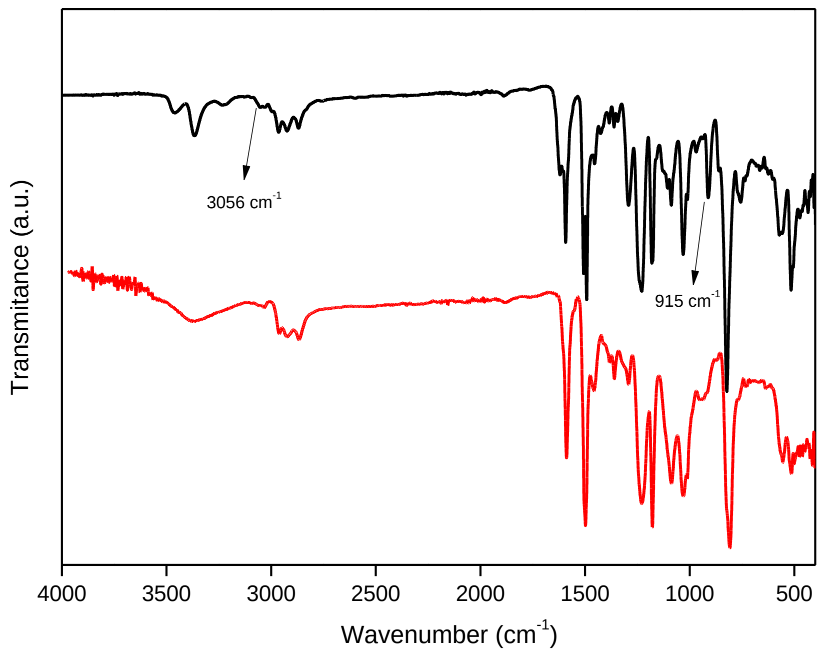

Figure S1. FTIR of uncured (black) and cured aero grade epoxy resin (red).

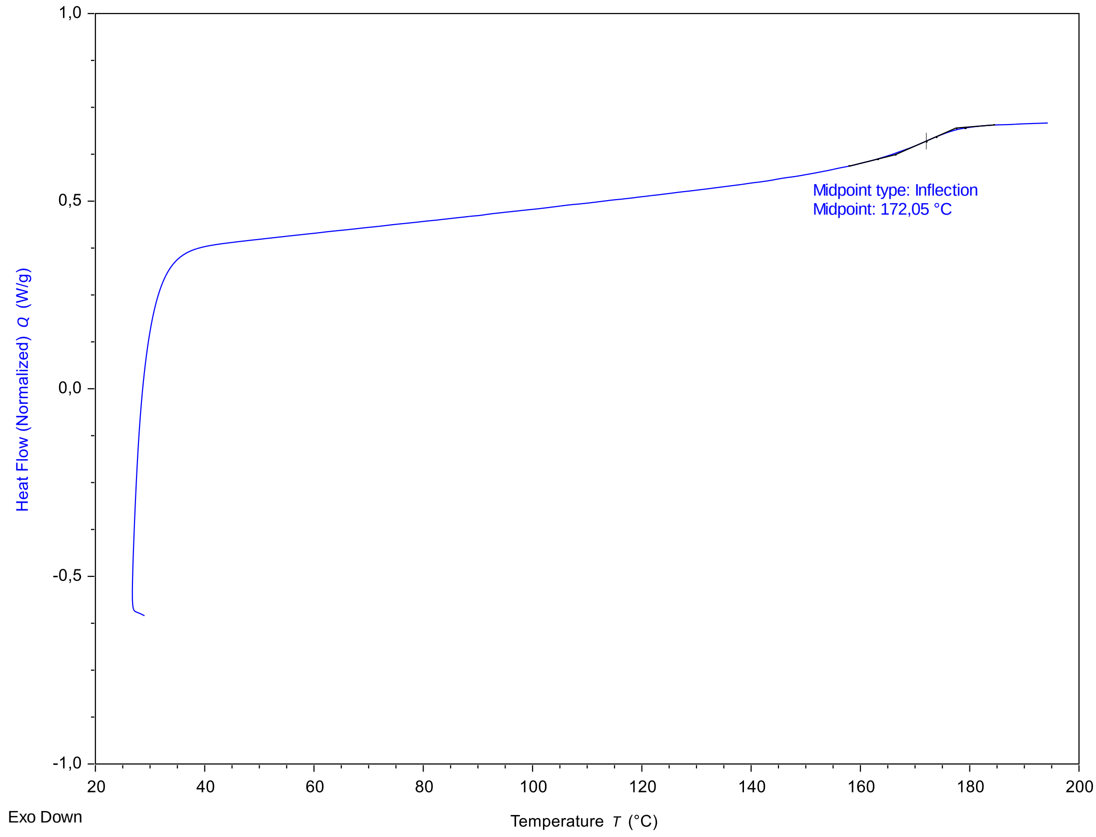

Figure S2. DSC thermogram for aero grade epoxy vitrimer, from where a Tg value of 172 °C was determined.

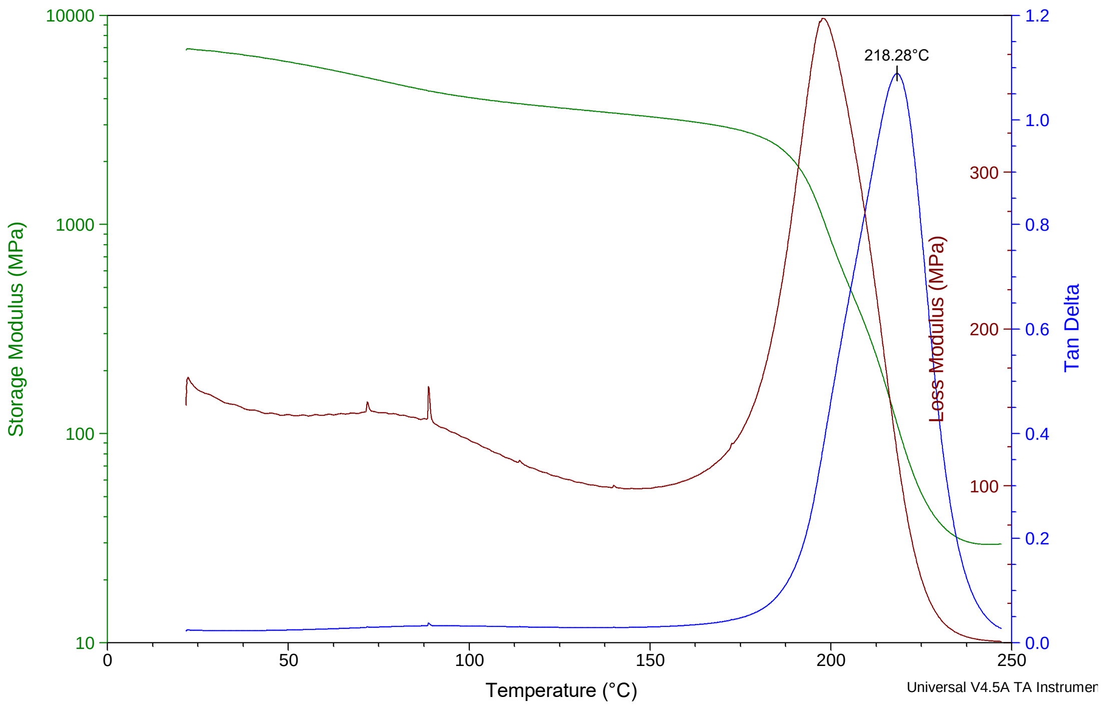

Figure S3. DMA curve obtained for RTM6, representing storage modulus, loss modulus and tan delta versus temperature. Tg = 218 °C was determined from the maximum of tan delta.

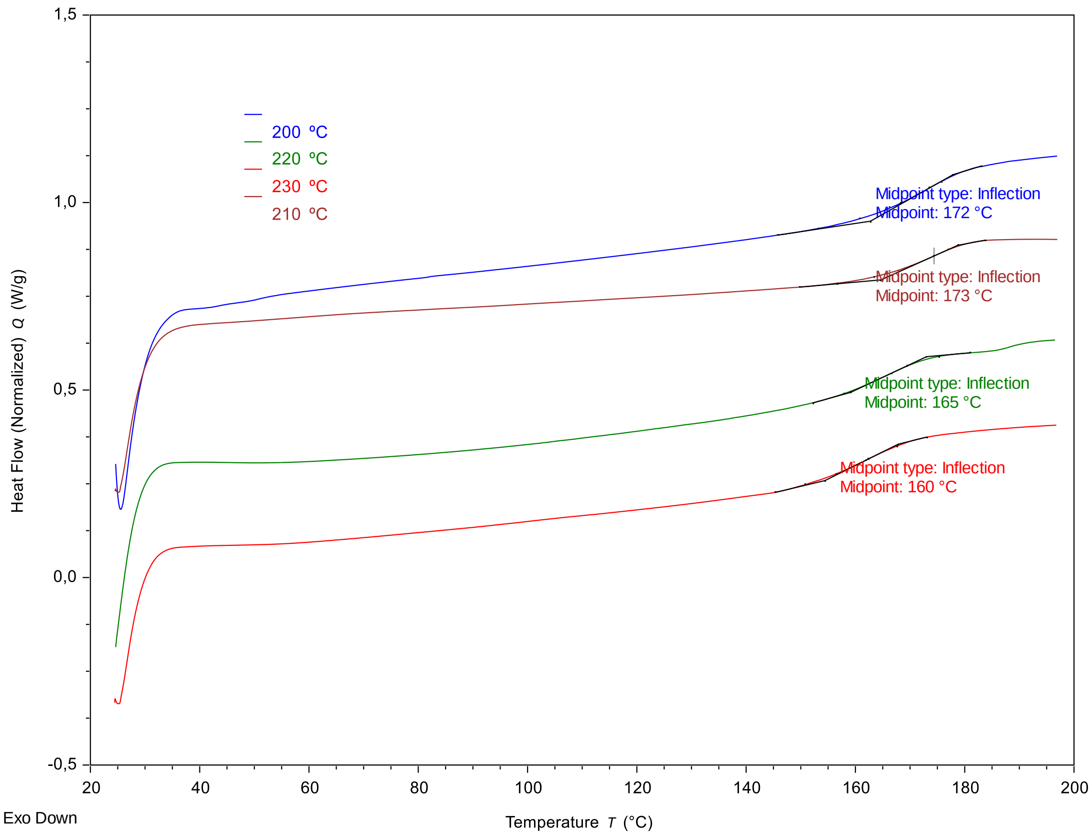

Figure S4. DSC thermograms of aero grade epoxy vitrimer after aging 20 min in ari atmosphere at 200 °C, 210 °C, 220 °C and 230 °C.

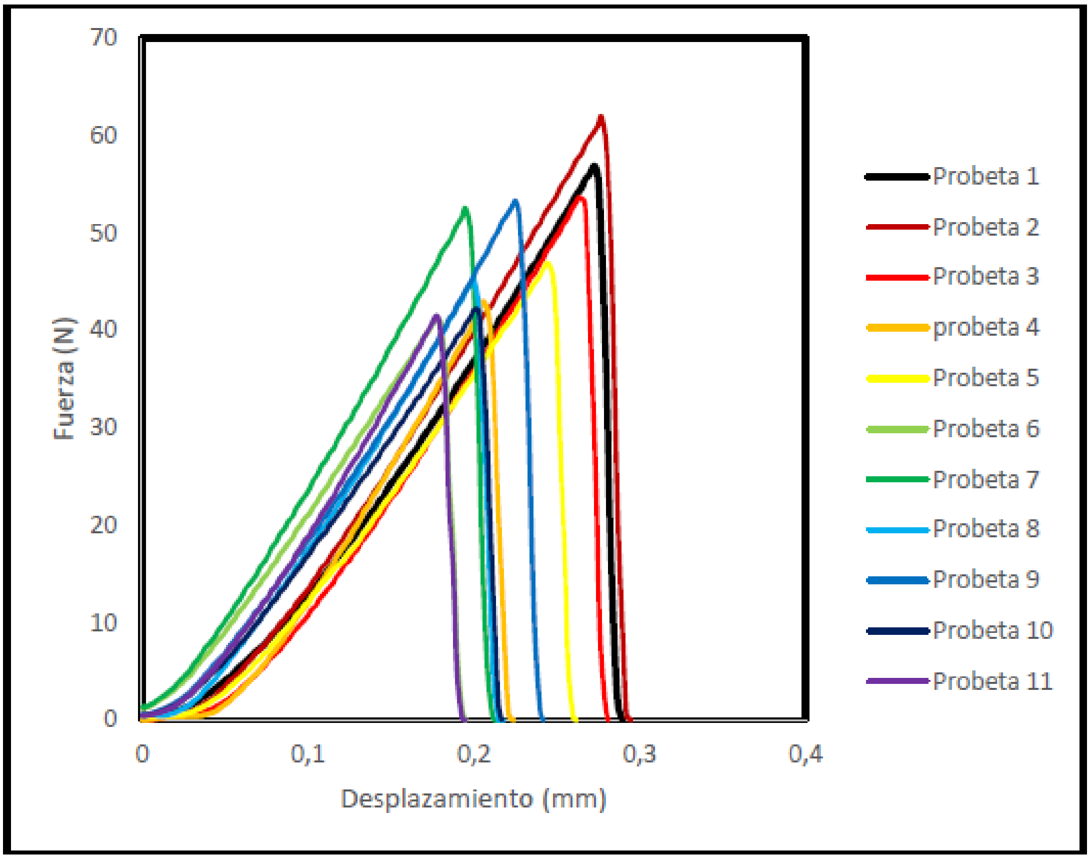

Figure S5. Force-displacement graphs of epoxy vitrimer.

Table S1. Values of the stress intensity factor K_Q for all tested materials.

Specimen	K_Q ($MPa.m^{1/2}$)
1	1.05
2	1.10
3	1.05
4	1.04
5	1.05
6	1.00
7	1.00
8	0.94
9	1.11
10	0.85
11	1.04
Average	1.02
S.D.	0.07

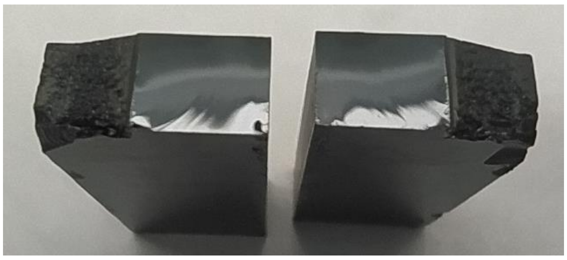

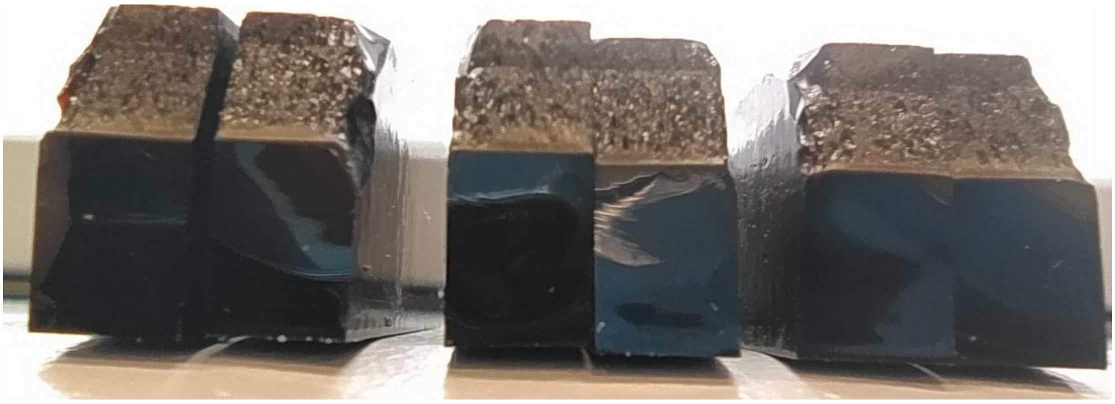

Figure S6. Fractured surfaces of tested aero grade epoxy vitrimer.

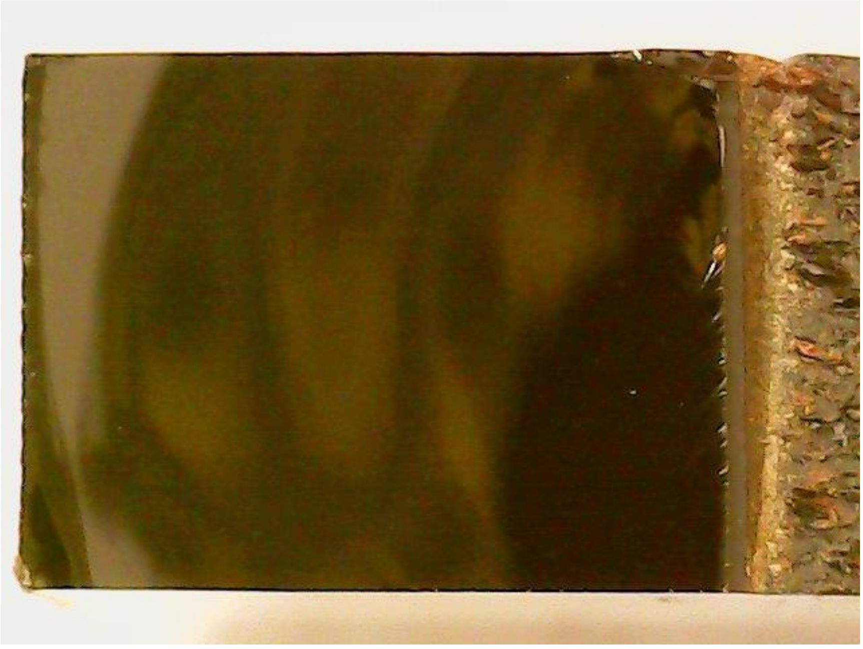

Figure S7. Detailed fracture surface analysis after failure. Smooth fracture surface.

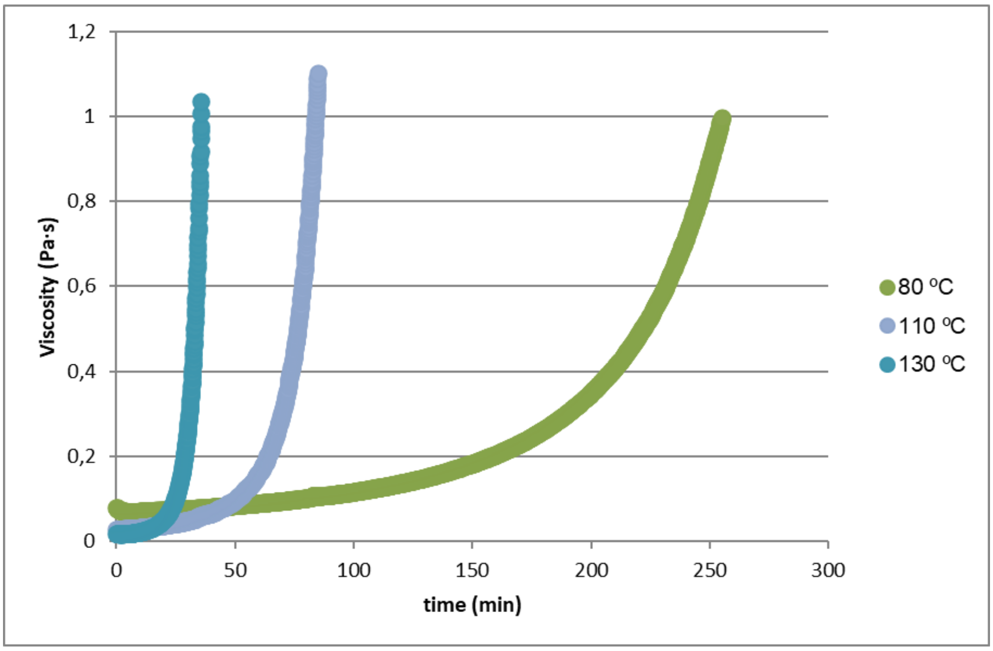

Figure S8. Viscosity evolution of aero grade epoxy resin at 80 ºC, 110 ºC and 130 ºC.

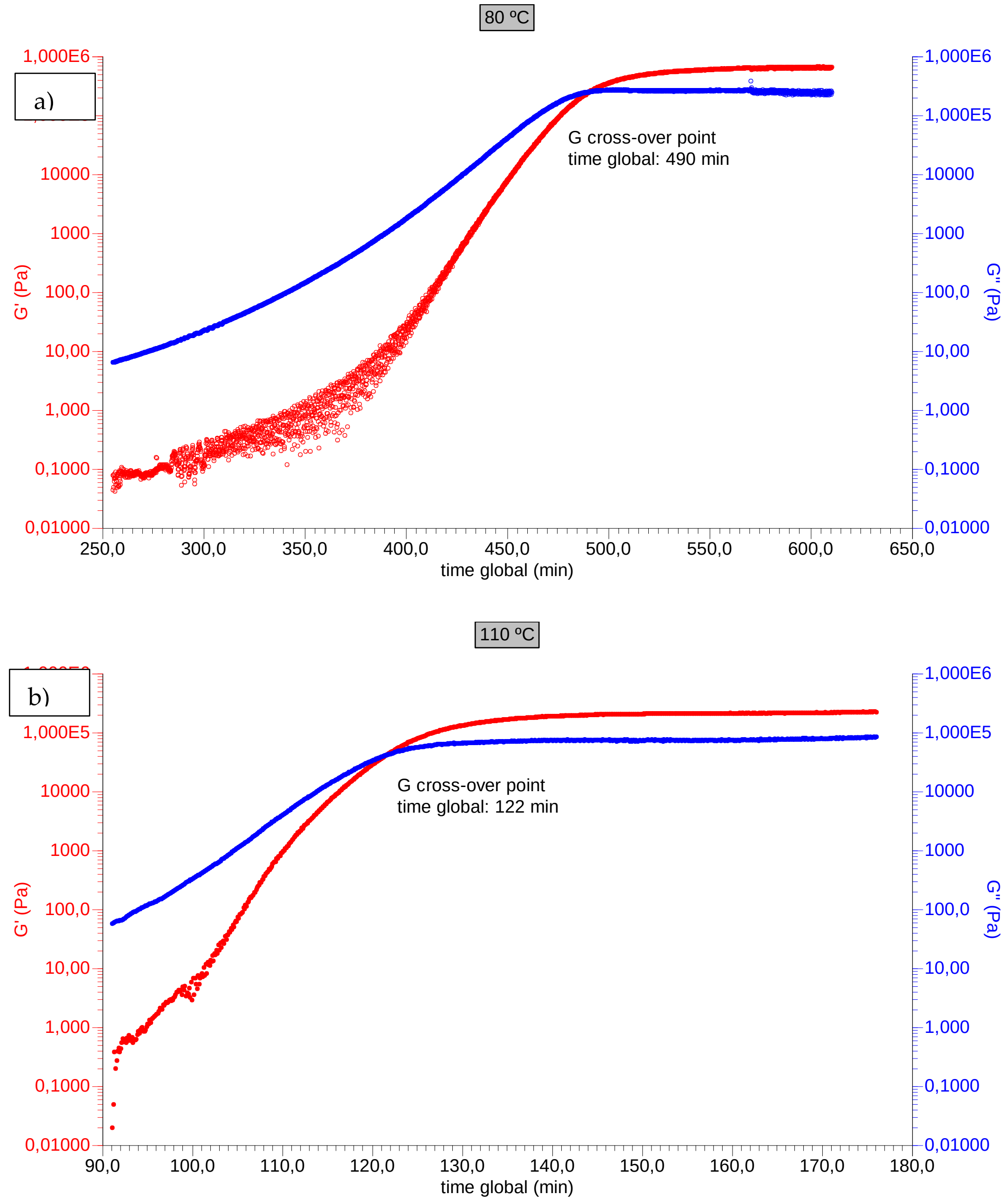
80 ºC
a)
G cross-over point
time global: 490 min
G' (Pa)
G'' (Pa)
time global (min)
1,000E6
1,000E5
10000
1000
100,0
10,00
1,000
0,1000
0,01000
250,0
300,0
350,0
400,0
450,0
500,0
550,0
600,0
650,0
110 ºC
b)
G cross-over point
time global: 122 min
G' (Pa)
G'' (Pa)
time global (min)
90,0
100,0
110,0
120,0
130,0
140,0
150,0
160,0
170,0
180,0

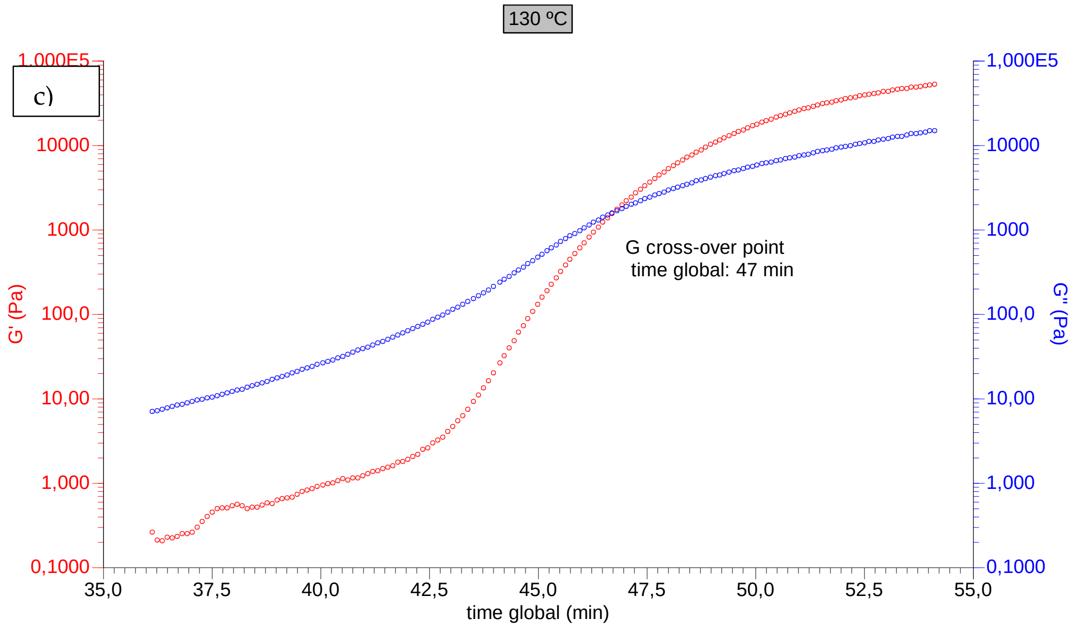

Figure S9. Gel point at 80 °C (a), 110 °C and 130 °C determined as the crossover point G′ = G′′.

Table S2. Enthalpy of cure, Tg of un-cured resin, onset curing temperature and exothermic temperature of aero grade epoxy vitrimer and reference RTM6 resin.

		Standard DSC parameters			
**Reference**	**Curing Ramp (°C/min)**	**Tg midpoint (°C)**	**Entalphy (J/g)**	**T peak (°C)**	**T onset (°C)**
Aero grade epoxy vitrimer	2.5	−19	529	159	131
RTM6	2.5	−18	534	201	178

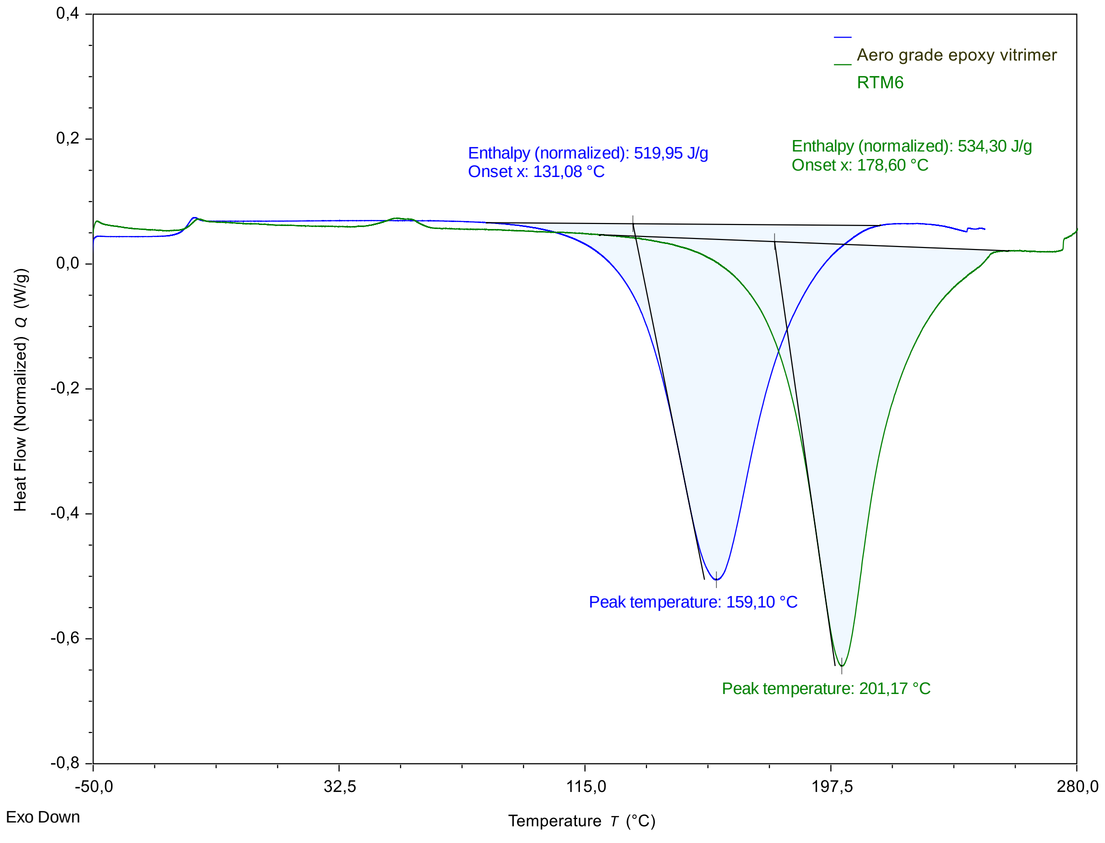

Figure S10. Dynamic DSC curves obtained at 2.5 °C/min for aero grade epoxy vitrimer (blue) and reference RTM6 resin (green).

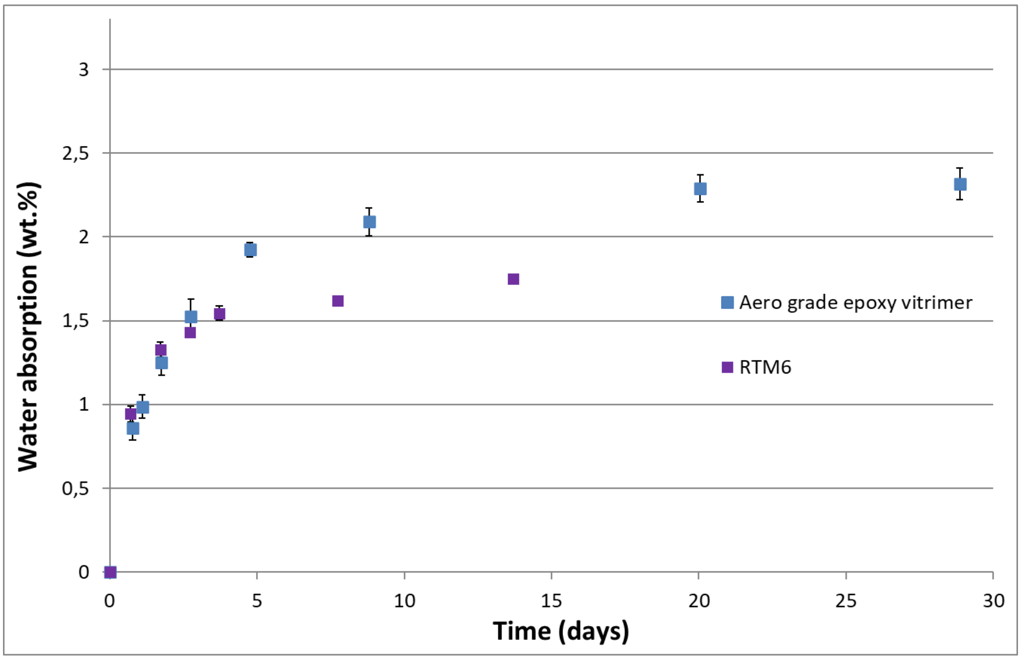

Figure 11. Water uptake of aero grade epoxy vitrimer.

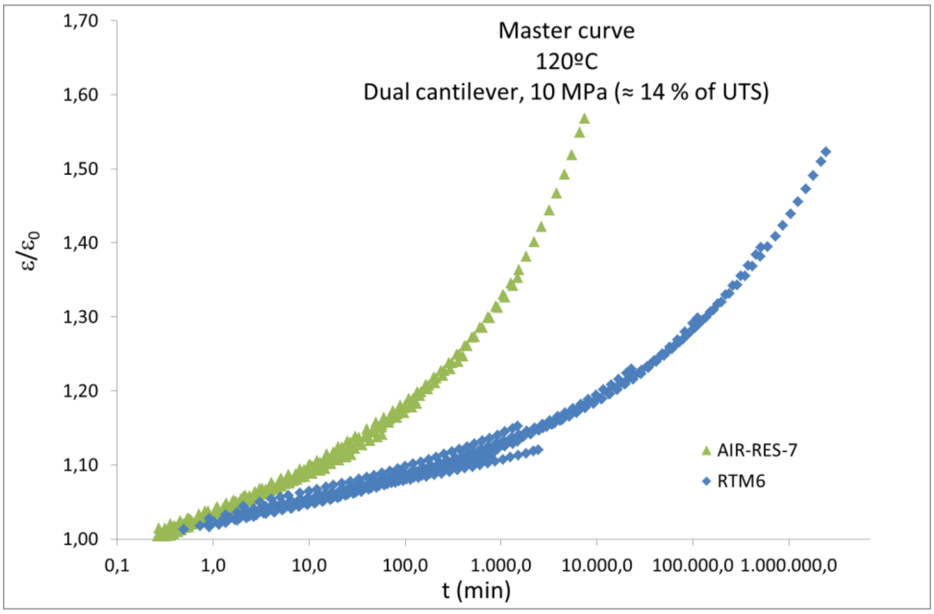

Figure S12. Creep strain master curve at 120 °C obtained by horizontal shifting of creep data at different temperatures.

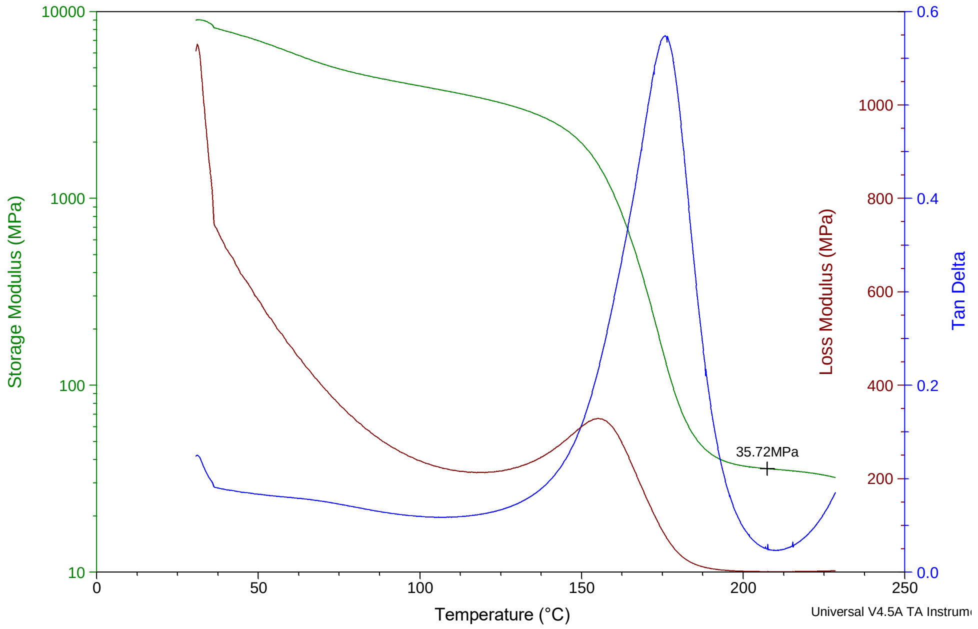

Figure S13. DMA curve obtained for thermoformed epoxy vitrimer, representing storage modulus, loss modulus and tan delta versus temperature. Tg = 175 °C was determined from the maximum of tan delta.

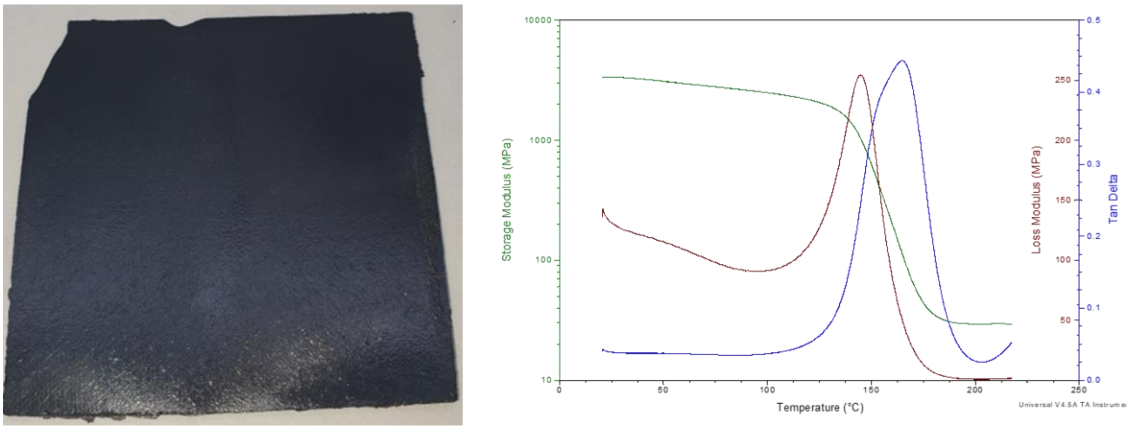

Figure S14. (Left) reprocessed aero grade epoxy vitrimer. (Right) DMA plot of reprocessed aero grade epoxy vitrimer.
